# Angioma serpiginosum: a case report and review of the literature

**DOI:** 10.1186/s13052-019-0644-8

**Published:** 2019-04-27

**Authors:** Andrea Diociaiuti, Mario Cutrone, Roberta Rotunno, Rita De Vito, Iria Neri, Elisa Pisaneschi, May El Hachem

**Affiliations:** 1 Dermatology Unit, Bambino Gesù Children’s Hospital, IRCCS, Piazza Sant’Onofrio, 4, 00165 Rome, Italy; 2 Paediatric Unit, Ospedale dell’Angelo, Mestre, Italy; 3 Pathology Unit, Bambino Gesù Children’s Hospital- IRCCS, Rome, Italy; 4 Division of Dermatology, University of Bologna, Bologna, Italy; 5 Molecular Genetics Laboratory, Bambino Gesù Children’s Hospital, IRCCS, Rome, Italy

**Keywords:** Angioma serpiginosum, WT-1, Dermoscopy

## Abstract

**Background:**

Angioma serpiginosum is a rare vascular anomaly whose pathogenesis is still unknown. It is characterized by the onset of vascular reddish macules and papules during childhood, lesions are usually monolateral with a linear serpiginous pattern. It is rarely associated with extracutaneous findings. This entity has not yet been included in the classification of the International Society for the Study of Vascular Anomalies.

**Case presentation:**

We describe the first Italian report of angioma serpiginosum with a congenital symmetrical presentation. The patient had a further extension of macules during puberty involving both of the soles. No extracutaneous manifestations were present. Diagnosis was confirmed with dermoscopy and light microscopy that revealed the typical clusters of dilated, thickened and PAS+ capillaries in the upper dermis. Moreover, Immunohistochemistry showed positive WT-1 staining. Genetic analysis with next generation sequencing did not detected any mutation.

**Conclusions:**

Our patient presented a peculiar symmetrical and planar extension with a serpiginous linear pattern. The proliferative nature of this condition has been widely discussed in literature. In our case immunohistochemistry was positive for Wilms tumor-1, a new endothelial marker expressed during angiogenesis in reparative processes and endothelial tumors.

Clinical evolution, histological and immunohistochemical findings suggest that angioma serpiginosum should be considered as a vascular proliferation. For these reasons we think it should be included in the international classification as a tumor.

## Background

Angioma serpiginosum (AS) was first described by Hutchinson in 1889 [1] as a “serpiginous or infective nevus” and then named by Crocker in 1894 [2]. This entity was distinguished by Frain-Bell from purpuric dermatoses and other conditions [3]. It is usually a sporadic condition; however familial cases have been reported with an autosomal dominant inheritance. Its pathogenesis remains unclear and no genetic mutation has been identified till now.

AS occurs at all ages, more frequently in childhood, with a female/male ratio of 9:1 [4]. This entity is not yet included in the classification of the vascular anomalies [5].

AS is characterized by the onset of vascular reddish macules and papules, grouped in a linear, serpiginous or gyrate pattern, sometimes on an erythematous skin [6, 7]. The serpiginous appearance is due to peripheral extension together with clearing of the central part of the vascular anomaly. The lesions are more frequently asymmetric and localized on the lower limbs and buttocks, but may affect other parts and rarely spread all over the body [7]. Some authors report a linear distribution resembling Blaschko lines and suggesting a type I mosaicism [6–10]. AS is asymptomatic and spreads usually within few years, but further extension is possible also later [4] and multiple recurrences are possible [11]. Partial spontaneous regression may occur resulting in cutaneous atrophy [4]. This disorder has a benign course, however, extracutaneous manifestations (ocular and neurological) and psychological distress are reported [12, 13], but no increased risk for neoplasm has been described. We describe the first Italian boy affected by AS with a peculiar symmetrical distribution and immunohistochemistry staining positive for Wilms tumor-1 (WT-1).

## Case presentation

**Fig. 1:**
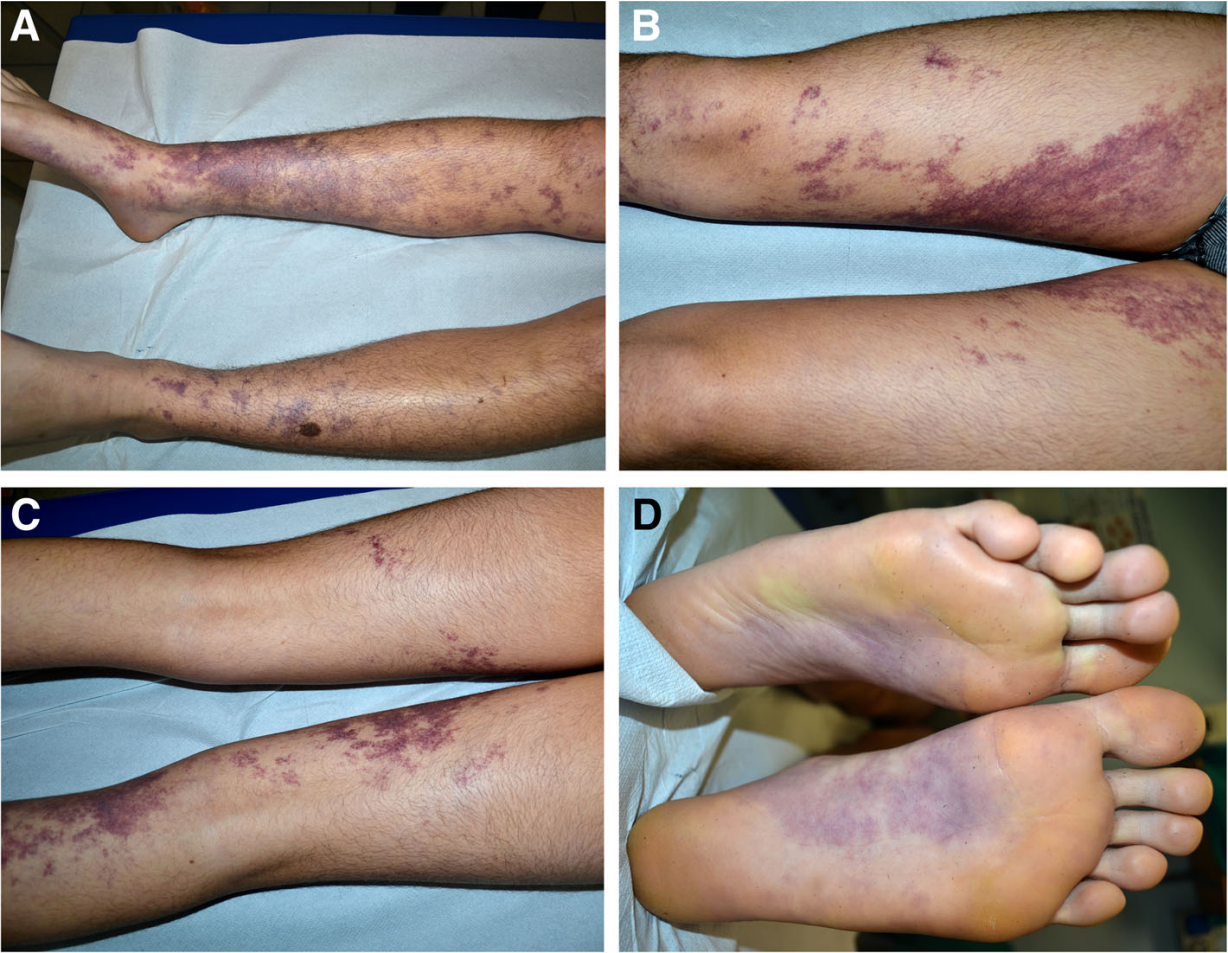
Asymptomatic purple maculopapular lesions, with a symmetrical serpiginous linear pattern along both the lower limbs (**a**,**b**,**c**) and soles (**d**)

**Fig. 2:**
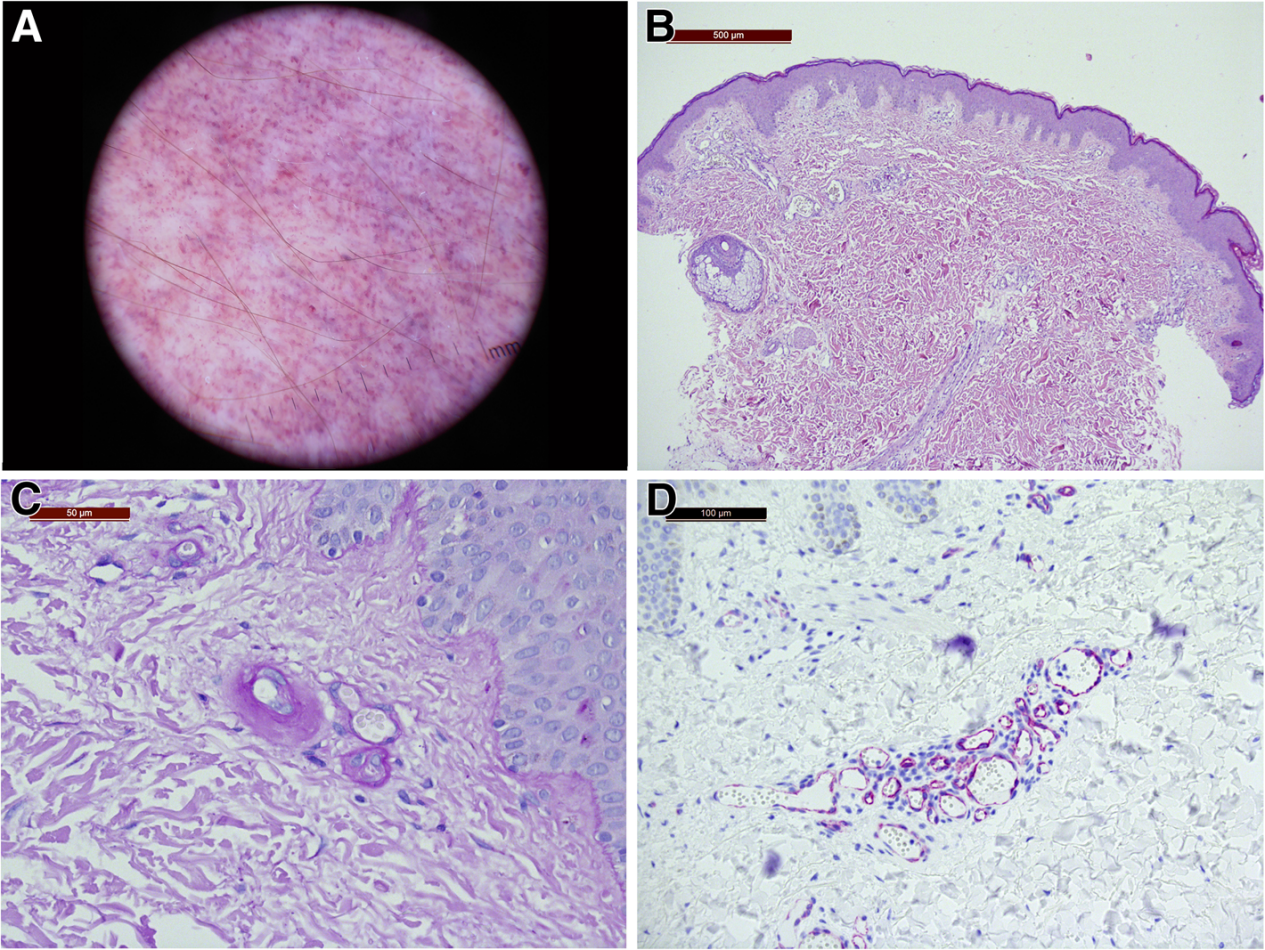
Dermoscopy shows parallel structures, red lagoons and globular elements (10x magnification) (**a**); light microscopy revealed clusters of dilated, thickened capillaries in the upper dermis composed of flattened endothelial cells (**b**); Periodic acid-Schiff (PAS) stain-positive deposits around the affected blood vessels (**c**); immunohistochemistry staining showed positive WT1 (**d**)

We report the case of a male child born at term from spontaneous delivery after an uncomplicated pregnancy. At birth, the newborn presented a vascular red macule on the right leg. At the age of 8, the lesions extended symmetrically to both lower limbs. The patient has been regularly followed by the paediatrician and never presented extracutaneous signs or symptoms. He has been referred to our vascular anomalies reference center at 14 years of age due to the increasing in color intensity of the lesions and the recent onset of new macules on the left forearm. Physical examination showed asymptomatic purple maculopapular lesions, with a symmetrical serpiginous linear pattern along both the lower limbs including the soles (Fig. 1). Other reddish pale macules were also present with a linear distribution on the left forearm and hand dorsum. No extracutaneous signs were present and no aesthetical complaint was expressed by the patient. Family history was negative. A dermoscopic examination revealed a parallel ridge pattern, sharply-demarcated red lagoons and pin-point and irregular globular elements (Fig. 2a). Based on history, and clinical and dermoscopic features, AS was suspected. The patient underwent a skin biopsy and a blood sampling for histology and molecular testing. Light microscopy revealed clusters of dilated, thickened and PAS+ capillaries in the upper dermis composed of flattened endothelial cells and pericytes (Fig. 2b, c). These features were observed in the absence of inflammatory infiltrate, erythrocytes extravasation and hemosiderin deposits, thus confirming the diagnosis of AS. Immunohistochemistry staining showed positive CD31, CD34, and Wilms tumor-1 (WT-1) and negative D2–40 and Glut-1 (Fig. 2d). No mutation was identified on the skin biopsy and peripheral blood, using MiSeq® sequencing platform (Illumina), with a specific panel for vascular malformations.

## Discussion and conclusions

AS is a rare vascular disorder, which over the years, has been alternatively considered as a nevoid vascular malformation or a vascular neoplasm [6].

The condition is asymptomatic and usually affects female patients, starting in childhood or early adolescence. Lesions are typically unilateral and located on the extremities with a predilection for lower limbs and buttocks [6]. However, any anatomic site, including palms and soles, can be involved. In the literature three cases with monolateral plantar area involvement have been described [7, 14–16]. Other few cases were characterized by a disseminated cutaneous distribution together with extracutaneous manifestations [12, 15, 17].

Our patient is male; he manifested macules since birth with a progressive serpiginous bilateral and symmetrical evolution, involving the lower limbs and the soles. The lesions were stable until the age of 14 years, when they spread to the upper arm. Dermoscopic features were similar to those reported in literature and they are correlated to the capillary dilation in the upper dermis [18–20]. Multiple red lagoons and pigment network in the absence of linear vessels are the most frequently observed alterations [21]. Reflectance confocal microscopy also displays the multiple dilated vascular spaces perpendicularly arranged to the epidermis in the superficial dermis. [22].

Pathogenesis of AS remains still unknown. Although most cases are sporadic, both autosomal dominant and X-linked dominant inheritance have been suggested. Accounting a female preponderance and progression of lesions in pregnancy, raised levels of estrogens have been postulated in the etiology, however, further studies refuted this hypothesis by the absence of estrogen-progesterone receptors [23]. Neumann et al. hypothesized an abnormal response to cold together with other unknown factors [24].

The proliferative or malformative nature of this entity has been widely discussed in the literature [25]. Histologically, the proliferation of endothelial cells and new formation of capillaries, without other alterations, have been considered the origin of the disease. These features and the absence of inflammation, erythrocyte extravasation and hemosiderin deposition clearly distinguish AS from purpuric dermatoses and acquired capillary malformation. Very few cases of acquired port-wine stain, also known as Fegelers syndrome, have been reported in literature, but they are unilateral on the head and neck, and characterized by a lower degree of proliferation [26]. Thus, some authors suggested classifying AS as a benign vascular tumor. In our patient, immunohistochemistry was positive for WT1 protein. Even if WT1 is not a causative gene for vascular anomalies, this new endothelial marker is expressed during angiogenesis in reparative processes or in benign and malignant endothelial tumors. WT1 has been studied in 126 vascular lesions (64 tumors, and 61 malformations) resulting positive in 100% vascular tumors and negative in 58 out of 61 (95.1%) vascular malformations [27–29]. Similar results have been published by Trindade et al. in a series of 117 vascular neoplasm that showed positive expression of WT1, whereas all vascular malformations were negative with the exception of arteriovenous malformations [30].

Genetic analysis, using the panel for vascular malformations containing all genes published in the last ISSVA classification [31], did not detect any mutation in our patient. Gunnar Houge described a deletion encompassing the PORCN gene in a four-generation family affected with AS, but, as clearly outlined by Happle, this family was affected by focal dermal hypoplasia [32, 33].

Treatment is indicated at any age when AS causes psychological discomfort to the patient. 532 nm potassium titanyl phosphate (KTP) laser ad pulsed dye laser (PDL) have been successfully used in AS with excellent response [34, 35] (Table 1). In our case no treatment has been performed because the patient does not complain esthetical damage.

**Table 1:** Recommendations for daily practice

When AS should be suspected?	The appearance after birth of vascular reddish macules and papules, grouped in a linear, serpiginous or gyrate pattern
When AS should be sent to the dermatologist?	Patient should be sent for diagnosis confirmation
How to confirm diagnosis of AS?	AS should be confirmed with dermoscopy and biopsy
What should be searched after the diagnosis of AS?	Ocular and neurological manifestations should be searched
Which is the indication to treat AS?	AS should be treated if the patient displays psychological discomfort

In our opinion, on the basis of the clinical evolution, histological and immunohistochemical findings, AS should be considered as a vascular tumor, and, anyway, it should be inserted in the ISSVA classification.

## Abbreviations

AS: Angioma serpiginosum
WT-1: Wilms tumor-1
